# Cost-effectiveness of seven-days-per-week rehabilitation schedule for acute stroke patients

**DOI:** 10.1186/s12962-023-00421-3

**Published:** 2023-02-01

**Authors:** Yasuhiro Morii, Kagari Abiko, Toshiya Osanai, Jiro Takami, Takumi Tanikawa, Kensuke Fujiwara, Kiyohiro Houkin, Katsuhiko Ogasawara

**Affiliations:** 1 Center for Outcomes Research and Economic Evaluation for Health, National Institute of Public Health, 2-3-6 Minami, Wako, Saitama Japan; 2 Faculty of Health Sciences, Hokkaido University, N12-W5, Kita-Ku, Sapporo, Hokkaido Japan; 3 Department of Rehabilitation Medicine, Sapporo Azabu Neurosurgical Hospital, N22-E1, Higashi-Ku, Sapporo, Hokkaido Japan; 4 Department of Rehabilitation Medicine, Hokkaido University Hospital, N15-W7, Kita-Ku, Sapporo, Hokkaido Japan; 5 Department of Neurosurgery, Graduate School of Medicine, Hokkaido University, N15-W7, Kita-Ku, Sapporo, Hokkaido Japan; 6 Department of Rehabilitation, Nishi Sapporo Hospital, 5-1, Yamanote 3-2, Nishi-Ku, Sapporo, Hokkaido Japan; 7 Faculty of Health Sciences, Hokkaido University of Science, 4-1, Maeda 7-15, Teine-Ku, Sapporo, Hokkaido Japan; 8 Graduate School of Commerce, Otaru University of Commerce, 5-21, Midori 3, Otaru, Hokkaido Japan

**Keywords:** Acute stroke, Rehabilitation, Cost-effectiveness, Cost utility analysis

## Abstract

**Background:**

Rehabilitation is an essential medical service for patients who have suffered acute stroke. Although the effectiveness of 7-days-per-week rehabilitation schedule has been studied in comparison with 5- or 6-days-per-week rehabilitation schedule, its cost-effectiveness has not been analyzed. In this research, to help formulate more cost-effective medical treatments for acute stroke patients, we analyzed the cost-effectiveness of 7-days-per-week rehabilitation for acute stroke from public health payer’s perspective, and public healthcare and long-term care payerʼs perspective in Japan.

**Methods:**

Cost-effectiveness of 7-days-per-week rehabilitation for acute stroke patients was analyzed based on the result from a previous study using a Japanese database examining the efficacy of 7-days-per-week rehabilitation. Cost utility analysis was conducted by comparing 7-days-per-week rehabilitation with 5- or 6-days-per-week rehabilitation, with its main outcome incremental cost-effectiveness ratio (ICER) calculated by dividing estimated incremental medical and long-term care costs by incremental quality-adjusted life years (QALY). The costs were estimated using the Japanese fee table and from published sources. The time horizon was 5 years, and Markov modeling was used for the analysis.

**Results:**

The ICER was $6339/QALY from public health payer’s perspective, lower than 5,000,000 Yen/QALY (approximately US$37,913), which was the willingness-to-pay used for the cost-effectiveness evaluation in Japan. The 7-day-per-week rehabilitation was dominant from public healthcare and long-term care payerʼs perspective. The result of sensitivity analysis confirmed the results.

**Conclusion:**

The results indicated that 7-days-per-week rehabilitation for acute stroke rehabilitation was likely to be cost-effective.

**Supplementary Information:**

The online version contains supplementary material available at 10.1186/s12962-023-00421-3.

## Background

Rehabilitation is an essential medical service for patients recovering from acute stroke, and the effectiveness of stroke rehabilitation has been reported in the literature [1–4]. The American Heart Association and American Stroke Association have stated the importance of early initiation of stroke rehabilitation [5]. Some studies consider the relationship between frequency of stroke rehabilitation and clinical outcomes such as length of stay [6–8]. Matsui et al. reported that being hospitalized on Friday would make post-stroke severity significantly worse, indicating that difference in treatments between weekdays and weekdays could affect patient outcomes [9]. Kinoshita et al. compared a group of acute stroke patients using the Japan Rehabilitation Database (N = 3072) who utilized rehabilitation services 7 days per week, with another group comprising those who utilized these services for 5 or 6 days [10]. The results showed that patient severity was significantly lower in the group with 7-days-per-week schedule even after adjustment by related parameters, and therefore, better outcomes can be expected.

In Japan, the importance of considering cost-effectiveness of medical services is being realized, and in 2019, the cost-effectiveness evaluation commenced [11]. In a study by Murayama et al. in 2011, only 4 out of 25 subject hospitals in Japan were found to provide acute stroke patients with rehabilitation 7 days a week [12]. It is possible that, currently, such hospitals are still not in majority. It is necessary to evaluate the cost-effectiveness of 7-days-per-week rehabilitation for acute stroke patients and consider optimal service fee in the national fee table to consider promoting that rehabilitation schedule. However, no study has considered its cost-effectiveness compared with a 5- or 6-days-per-week schedule. In this study, the cost-effectiveness of a 7-days-per-week rehabilitation schedule for acute stroke patients is analyzed to help formulate more cost-effective rehabilitation for acute stroke patients.

## Materials and methods

### Scheme and outcomes

The subjects were a cohort of 1000 hypothetical patients who had experienced an acute stroke in Japan. The patient characteristics were assumed to be as follows: 75 years old, less than 1 day to admission after onset, having NIHSS (National Institutes of Health Stroke Scale) scores of 5–6 on average at admission, with mRS scores of 3, 4, or 5 (physically severe) at admission. Approximately 70% had cerebral infarction, and approximately 40% were female. The patient characteristics were defined according to Kinoshita et al., the basis of the study analysis [10]. The study compared functional outcomes such as the rates of patients who were physically independent (mRS0-2) between the 7-day-per-week rehabilitation schedule and that of 5-or 6-day per week using Japanese Rehabilitation Database.

**Fig. 1:**
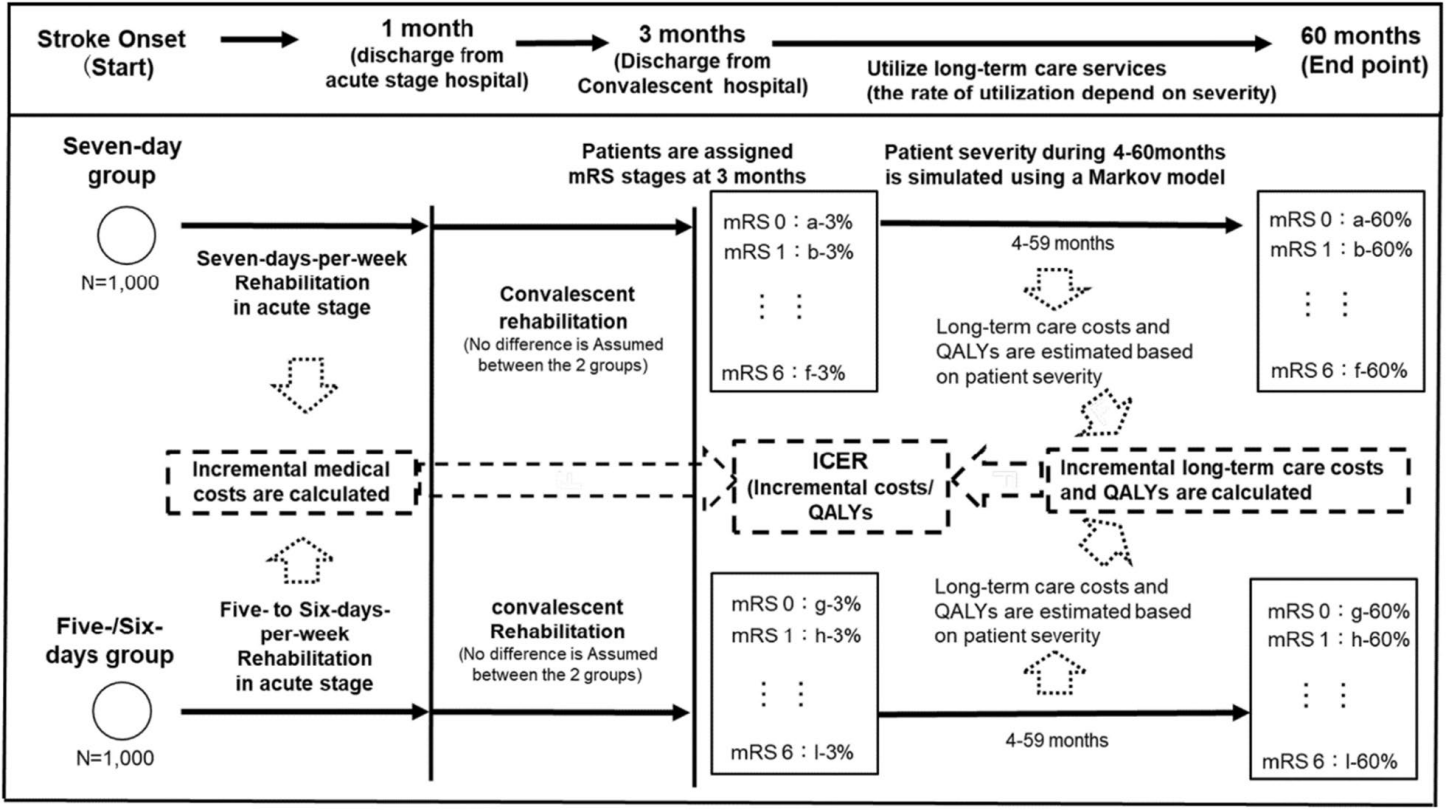
The design of this study. It shows the design of this study. The hypothetical subjects comprise 2 groups of acute stroke patients; one group takes rehabilitation 7 days per week for 30 days after onset, and the other, 5 days. It is assumed that those patients are admitted to acute hospitals and thereafter to convalescent hospitals. It is assumed that medical cost difference only arises from the frequency of rehabilitation in the acute stage, and the cost difference was analyzed. After the discharges, patients utilize long-term care according to severity, estimated using Markov model simulation (described in later section “Patient severity estimate”). Quality adjusted life years are also estimated from the estimated severity. Incremental cost-effectiveness ratio was calculated as a primary outcome for cost-effectiveness evaluation

One patient group used conventional rehabilitation services 5 or 6 days a week, and the other, for 7 days a week, for 30 days after the onset of stroke in acute hospitals (5-/6-day group and 7-day group, respectively). The two groups were compared in the cost utility analysis by a cohort-level simulation, for which effectiveness of the 7-days-per-week rehabilitation was based on the study by Kinoshita et al. [10]. The time horizon was five years after onset. The design of this study was shown in Fig. 1. In Japan, it was common for acute stroke patients to be admitted in an acute hospital, and after being discharged, in a convalescent hospital, for treatments such as rehabilitation. In the National fee schedule in Japan, additional fee for acute stroke rehabilitation can be charged for 30 days after onset [13]. Based on the information in the National fee schedule, it was assumed that the length of stay in acute hospitals in this study is 30 days, and that in convalescent hospitals is up to 90 days (i.e., 3 months) [14]. It was also assumed that the two groups differed in the frequency of rehabilitation services utilized in the acute stage, though there was no such difference in medical services they received in the convalescent stage [10]. From the 4th month, the patients utilized long-term care services according to their severity, which was described later section (“Long-term-care cost").

The primary outcome for the cost utility analysis was the incremental cost-effectiveness ratio (ICER), which was commonly used for health technology assessment in many countries such as UK [15] and Japan [11]. Quality-adjusted life years (QALY) was used as a measure of outcome. Calculated ICERs were evaluated with the willingness-to-pay of 5,000,000 yen/QALY (approximately US$37,913), in accordance with the one used in the cost-effectiveness evaluations in Japan [16].

In the Japanese medical system, universal rates are decided for each medical service, treatment, or drug in the national fee schedule [17]. The rate of co-payment differs depending on patient groups (e.g., 30% for most patients, 10% for patients aged 75 years or older, and no charge for welfare recipients), and the rest is paid by insurers and the government. In total, only 11% of the national medical expenditure is covered by patient co-payments. A large part is covered by the public budget. The Guideline for Preparing Cost-Effectiveness Evaluation in Japan recommends that health technology assessments be conducted from the public health payer’s perspective as a standard. This only includes public medical costs within the range of public healthcare insurance in Japan since decisions for price adjustments are made within that range in the Japanese cost-effectiveness analysis [11]. Long-term care services in Japan have also been administered to the universal system, and the universal rates are decided for each service. The guideline also states that public healthcare and long-term care payer’s perspective are acceptable when the effect of public long-term care costs is important. This perspective includes both public medical costs within the range of the public healthcare insurance in Japan and long-term care costs (i.e., costs for utilization of public long-term care services) within the range of public long-term care insurance. Since this was the case with acute stroke, by which patients had severe sequela, our analysis was based both on the public health payer’s perspective, and public healthcare and long-term care payerʼs perspective. The national fee schedule published in 2020 [13] was used. The costs and QALYs were discounted by 2% per year, according to the Japanese guideline [11]. After the analysis, the results were converted to US$ using the currency exchange rate as of January 9, 2023 (US$1 = 131.88 yen).

### Patient severity estimate

Patient severity was based on the modified Rankin Scale (mRS). Patient severity from the fourth to the 60th month was estimated using Markov modeling. The Markov model was a simulation model in which transition states and transition probabilities, as well as patients’ moves between the transition states at a Markov cycle are defined. The Markov model has been applied to simulations of patients with various diseases [18, 19] and with stroke patients [20, 21].

**Fig. 2:**
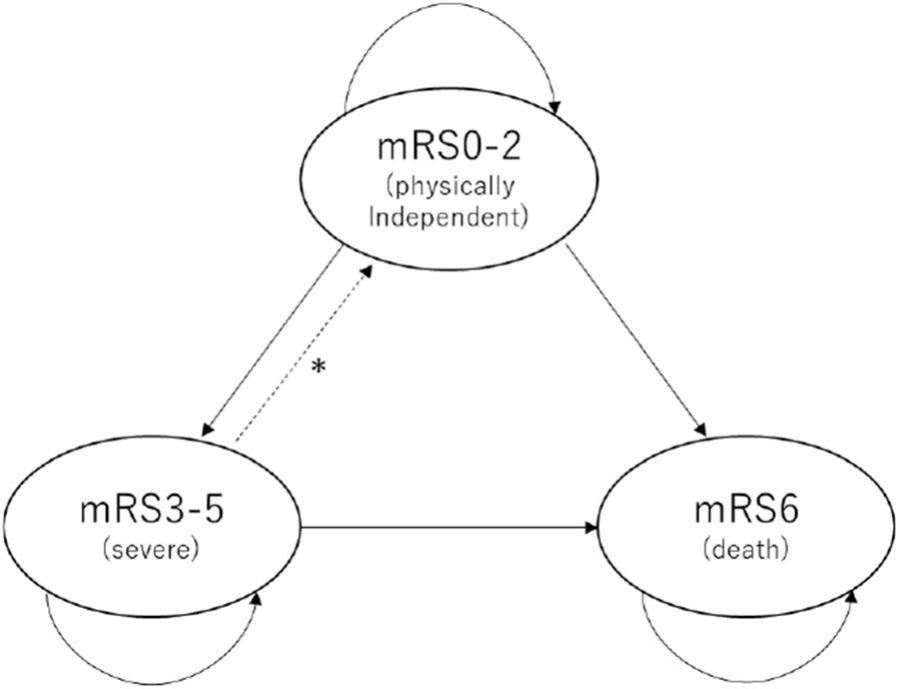
The structure of Markov model. It shows the structure of the Markov model. The model consisted of three health states: modified Rankin Scale (mRS) 0–2, mRS3-5, and mRS6. The transition from mRS3-5 to mRS0-2 (*) happens only in the first year

In this study, three states—mRS0-2 (functionally independent), mRS3-5 (disabled), and mRS6 (death)—were defined (Fig. 2), and the Markov cycle was 1 month.

The initial probabilities (mRS distribution at month 3) were mRS0-2: mRS3-5: mRS6 = 49.3%: 47.0%:3.9% in the 7-day group, and 37.6%: 58.5%: 3.9% in the 5-/6-day group. The initial probability of mRS0-2 in the 5-/6-day group was in line with the result of Kinoshita et al., and that of the intervention group was estimated by multiplying the result of the control group by the risk ratio for mRS0-2 (1.31 (95%CI: 1.20–1.43)) estimated from the odd ratio (1.62 (95%CI: 1.36–1.94)) taken from the study by Kinoshita et al. [10]. In the study by Kinoshita et al., the odds ratio was calculated from a model that made adjustments for age, sex, stroke subtype, time to admission after onset, NIHSS score, mRS score, each comorbidity, t-PA administration, operative treatment, daily rehabilitation time, and time to rehabilitation after admission. The proportion of mRS6 patients was assumed to be the same between the two groups at the initial distribution.

The transition probabilities were estimated by calibration in accordance with Xie et al. [22], which was based on Oxford Vascular Study [23], a study of long-term outcomes for stroke patients with a large number of samples (see Additional file 1 for details). The Life Table of Japan [24] was used for calibration. In the model, mortality was assumed by multiplying the mortality for general population at the corresponding age by disease-specific risk ratio of mortality for stroke patients, which was estimated by the calibration. It was also assumed that patients at mRS3-5 state could move to mRS0-2 state in the first year since that recovery was possible during the period. In addition, the transition probabilities after 5 years, used in the scenario analysis, were based on the parameters in the fifth year, adjusted with the corresponding age-specific mortality for general population. See Additional file 1 for details of the calibration.

After the simulation using the Markov model, patients were divided into finer mRS stages (mRS0-2 into mRS0, 1, 2, respectively, and mRS3-5 into mRS3, 4, 5, respectively) at each time cycle since differences in QALYs and long-term care costs could exist even within functionally independent patients or disabled patients. Patients at mRS0-2 and mRS3-5 stage were divided into finer mRS grades using the proportions of mRS0:1:2 = 30.5%:29.8%:39.6%, mRS3:4:5 = 37.5%:36.9%:25.6%, respectively, according to the proportion of patients by Hattori et al. [25], and those proportions were assumed not to change during the time horizon.

Based on the estimated patient severity, QALYs and long-term care cost estimates were analyzed, as explained later. R ver. 3.5.2 was used for the simulation.

### Medical cost

In this study, it was assumed that there was no difference in medical services provided to the 7-day and 5–6-day groups, except for the number of rehabilitation service units in the acute stage. No significant difference was found between the two groups in length of stay and treatments provided in the acute stage, such as administration of recombinant tissue plasminogen activator in the study by Kinoshita [10]. It was also assumed that medical costs after discharge from acute hospitals were not included in the analysis.

In the medical cost analysis, patients utilized rehabilitation services for 30 days after onset. The day of onset was randomly assigned to patients from Sunday to Saturday, because the number of rehabilitation days were affected by what day of the week the stroke occurred.

In the Japanese public medical system, the time required for rehabilitation services is 20 min per unit [11]. It was assumed that the number of rehabilitation units a patient utilizes per day was 4.3 [10]. The national fee for per-unit rehabilitation service is $18.6. An additional fee of $2.41 per unit was charged for rehabilitation during the first 30 days, and there is another fee of $3.62 per unit charged during the first 14 days [13], which is charged when a full-time (or equivalent) rehabilitation physician is engaged. Since there were no data on proportion of hospitals that provide rehabilitation for acute stroke patients 5–6 days a week, it was assumed that all the patients in the 5-/6-day group utilized rehabilitation services 5 days a week for a conservative cost estimate.

### Long-term-care cost

It was assumed that long-term-care costs differed depending on patient severity. The Japanese long-term care system divides patients with care needs into seven levels based on their severity: Support level 1–2 and Care Needs level 1–5 [26]. The higher the level, the severer a patient is. Patients with Support level needs are less severe than those with Care Needs level. First, the estimated patient severity in the form of mRS stages was transformed into severity in the form of long-term-care needs (the seven levels), according to a previous study [27] (Table 1). For example, to estimate long-term-care costs, patients with mRS1 disability were assigned to Support level 1. Patients with mRS4 were assigned to Care Needs level 2 or 3, and patients with mRS5 were assigned to Care Needs level 4 or 5, according to the proportion of the patients in each Care Needs level in the previous survey by the Ministry of Health Labor and Welfare [28] (i.e., Care level 2:3 = 60.8%:39.2%, Care level 4:5 = 61.0%:39.0%). The rate of service utilization was defined by patient severity under the premise that, the severer a patient, the more care services she/he is likely to be utilized (Table 1) [26]. Data on long-term-care costs were calculated according to a previous study by Yamaga and Ikeda, in which the costs were estimated based on a survey by the Ministry of Health Labor and Welfare by dividing the amount of total long-term expenditure at each Care Needs level by the number of patients in each Care Needs level [29] (Table 1).

**Table 1:** Utility, care needs level, utilization rate of long-term care services, and long-term care costs by mRS grade

	mRS0	mRS1	mRS2	mRS3	mRS4	mRS5	mRS6
QOL utility [25] (base case)	0.89 γ(61.0, 68.5)	0.797 γ(90.0, 113.0)	0.65 γ(77.1, 118.7)	0.588 γ(77.0, 131.0)	0.363 γ(24.7, 68.1)	0.092 γ(1.32, 14.4)	0 (0)
Care needs level [27]		Support level 1	Support level 2	Care level1	Care level 2 or 3*	Care level 4 or 5*	
Utilization rate of long-term care services [25]	0%	21.6%	91.5%	98.5%	1	1	0
Long-term care costs based on care needs level ($/month) [29]		219 γ(34.5, 0.2)	376 γ(36.4, 0.1)	806 γ(167.2, 0.2)	Level 2: 1090 γ(306.1, 0.2) Level 3: 1596 γ(655.6, 0.4)	Level 4: 1914 γ(517.3, 0.3) Level 5:2205 γ(686.3, 0.3)	

*Patients are assigned to the either of the 2 Care Level according to the proportion of the patients shown in Comprehensive Survey of Living Conditions [28] (Care level 2:3 = 60.8%:39.2%, Care level 4:5 = 61.0%:39.0%)

*mRS* modified Rankin Scale, *QOL* quality of life

Table 1 shows the parameter inputs and their distributions for probabilistic sensitivity analysis for each mRS grade used in the analysis.

### Utility estimation

The patients were assigned quality of life (QOL) utility according to their mRS grades at each time cycle (Table 1). Data on utility for each mRS stage was obtained from the study by Hattori, which was conducted on Japanese subjects [26]. The QOL utility collected from Japanese results is preferred in the Japanese guideline [11].

### Sensitivity and scenario analysis

To consider the uncertainty of cost-effectiveness, sensitivity analysis and scenario analysis were conducted. The sensitivity analysis consisted of one-way deterministic sensitivity analysis and probabilistic sensitivity analysis (PSA).

The parameters and the sensitivity ranges for one-way deterministic sensitivity analysis are shown in Table 2. The target parameters were efficacy of the intervention, mRS distribution in the same model states (mRS0:1:2 = 30.5%:29.8%:39.6%, mRS3:4:5 = 37.5%:36.9%:25.6% in the base case), utilization rates of long-term care services, rehabilitation fees, long-term care fees, the number of rehabilitation units provided per day.

**Table 2:** Ranges of parameters for sensitivity analysis and scenario analysis

Parameters	Lower case	Base case	Upper case	Source
Sensitivity analysis				
Efficacy of the intervention (mRS distribution at 90 days)	mRS0-2:mRS3-5:mRS6 = 45.0%:51.1%:3.9%	mRS0-2:mRS3-5:mRS6 = 49,3%:46,7%:3.9%	mRS0-2:mRS3-5:mRS6 = 53.8%:42.2%:3.9%	[10]
mRS distribution for severe patients (mRS3-5)	mRS3:4:5 = 100%:0%:0%	mRS3:4:5 = 30.5%:29.8%:39.6%	mRS3:4:5 = 0%:0%:100%	
mRS distribution for non-severe patients (mRS0-2)	mRS0:1:2 = 100%:0%:0%	mRS0:1:2 = 30.5%:29.8%:39.6%	mRS0:1:2 = 0%:0%:100%	
Utilization rates of long-term care services	− 10% from base case	Shown in Table 1	+ 10% from base case	
Rehabilitation fees	− 50% from base case		+ 100% from base case	
Long-term care fees	− 50% from base case		+ 100% from base case	
# of rehabilitation units per day	1 units	4.3 units	9 units	[10, 13]
Scenario analysis				
Discount rate (%/year)	0	2	4	[11]
Time horizon	10 years	5 years		

Target parameters for the PSA were the efficacy of 7-day rehabilitation, utility, and the number of rehabilitation units provided per day. For the efficacy of 7-day rehabilitation, the risk ratio was determined from the normal distribution (Normal (1.31, 0.05)) using the value calculated in the "Patient severity estimate" section. Utility and the long-term care costs were determined from gamma distribution (Table 1) for each patient. To estimate the distribution of long-term care costs, chronic care costs for Japanese stroke patients by Kamae et al., were used [30]. Regarding the medical costs, the number of rehabilitation units per day was determined from the normal distribution (Normal (4.3, 1.0)) [10]. The number of iterations for the PSA was 1000.

For the scenario analysis, analysis with 10-year time horizon was also conducted, and the discount rate was changed between 0 and 4% per year [11] (Table 2).

## Results

### Medical cost and long-term-care cost

The summary of results is shown in Table 3. In the base case, the average medical cost for rehabilitation at the acute stage per patient was $2928 for the 7-day group, and $2092 for the 5-/6-day group. Average long-term-care costs per patient for the 5 years were $29,286 and $31,646 for the 7-day group and the 5-/6-day group, respectively.

**Table 3:** Costs, gained QALYs, ICERs in the base case

Scenario	Medical cost ($US)	Long-term care cost ($US)	Total cost ($US)	Gained QALY	Public healthcare payerʼs perspective,	Public healthcare and long-term care payerʼs perspective
					ICER(US$/QALY)	ICER(US$/QALY)
7-Day schedule	2928	29,286	32,214	1.855	6339	Dominant
Δ From 5-/6-day schedule	836	− 2360	− 1524	0.132		
Five-/6-day schedule	2092	31,646	33,738	1.723		

From public healthcare and long-term care payerʼs perspective, the average total incremental cost per patient were $− 1524.

### Incremental QALYs

The average gained QALYs for the 5 years were 1.855, and 1.723 for the 7-day group and the 5-/6-day group, respectively, and the incremental QALY was 0.132 (Table 3).

### Incremental cost-effectiveness ratio

From public healthcare payerʼs perspective, ICER was $6339/QALY (Table 3). From public healthcare and long-term care payerʼs perspective, the 7-day group was dominant.

Table 3 shows the results of base case on medical cost, long-term care cost, QALY, and ICER.

### Sensitivity and scenario analysis

**Fig. 3:**
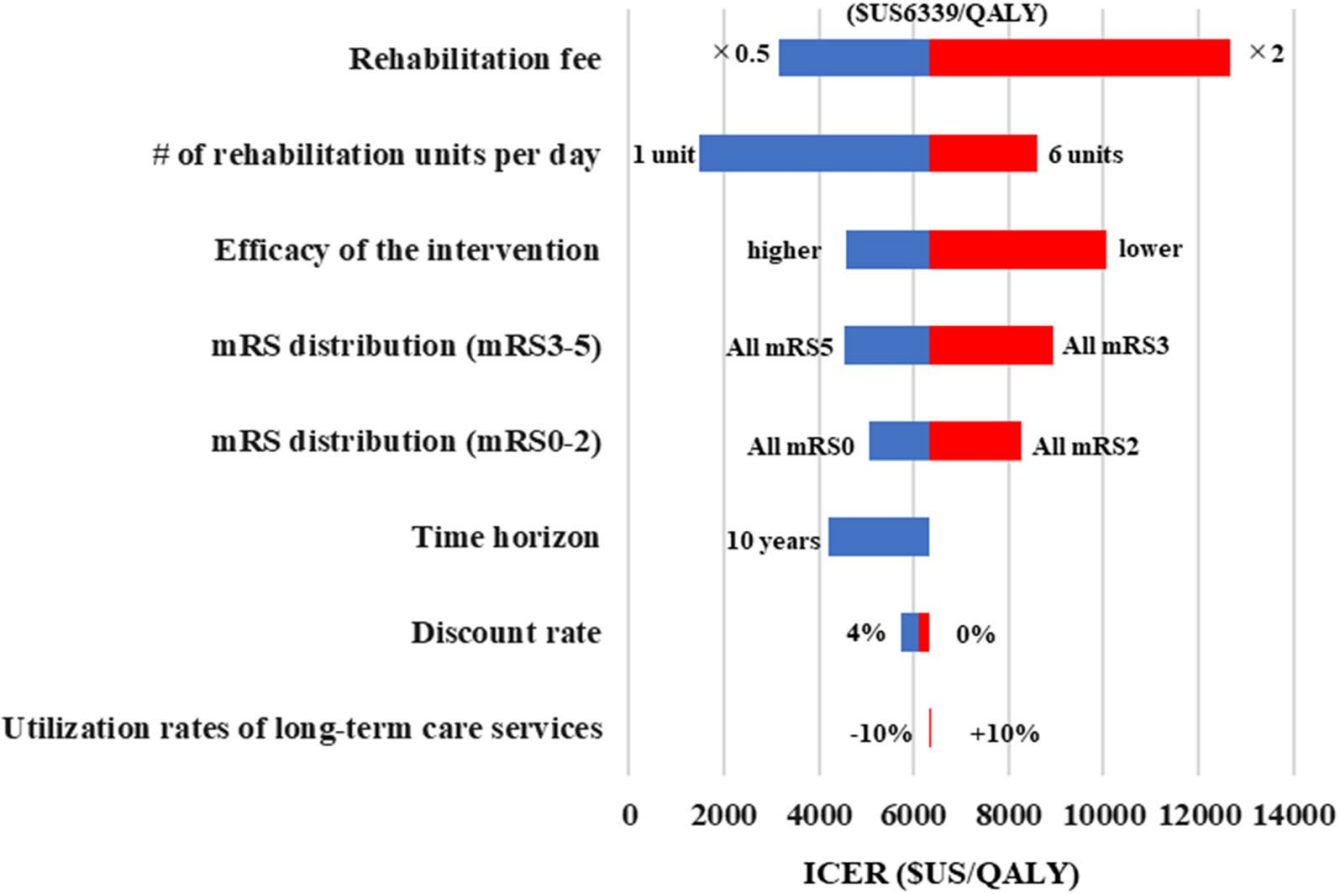
Results of deterministic sensitivity analysis and scenario analysis. It shows the results of one-way deterministic sensitivity analysis and scenario analysis

The results of sensitivity analysis and scenario analysis from public healthcare payerʼs perspective were shown in a tornado chart (Fig. 3). Rehabilitation fee had the most impact on the ICERs, which ranged from US$3169 to US$12,678/QALY, followed by the number of rehabilitation provided per day with ICERs ranging from US$1473 to US$8607/QALY.

ICER was $4210/QALY when the time horizon was 10 years.

The results of sensitivity analysis from public healthcare and long-term care payerʼs perspective were shown in Table 4. Seven-days-per-week rehabilitation stayed dominant even when the values of the parameters were changed.

**Table 4:** Results of sensitivity analysis and scenario analysis from public healthcare and long-term care payerʼs perspective

Parameter	Lower case			Base case			Upper case		
	ΔQALY	ΔCost ($)	($/QALY)	ΔQALY	ΔCost ($)	ICER ($/QALY)	ΔQALY	ΔCost ($)	ICER ($/QALY)
Efficacy of the intervention (risk ratio of mRS0-2 patients)	0.083	− 652	Dominant	0.132	− 1524	Dominant	0.182	− 2424	Dominant
mRS distribution (mRS3-5)	0.093	− 430	Dominant				0.184	− 3138	Dominant
mRS distribution (mRS0-2)	0.164	− 2000	Dominant				0.10	− 917	Dominant
Discount rate	0.138	− 1603	Dominant				0.148	− 1452	Dominant
Utilization rates of long-term care services	0.132	− 909	Dominant				0.132	− 1472	Dominant
Rehabilitation fee	0.132	− 689	Dominant				0.132	− 1943	Dominant
Long-term care service fee	0.132	− 344	Dominant				0.132	− 3885	Dominant
# of rehabilitation units per day	0.132	− 1654	Dominant				0.132	− 1234	Dominant
Time horizon (10 years)							0.20	− 1565	Dominant

**Fig. 4:**
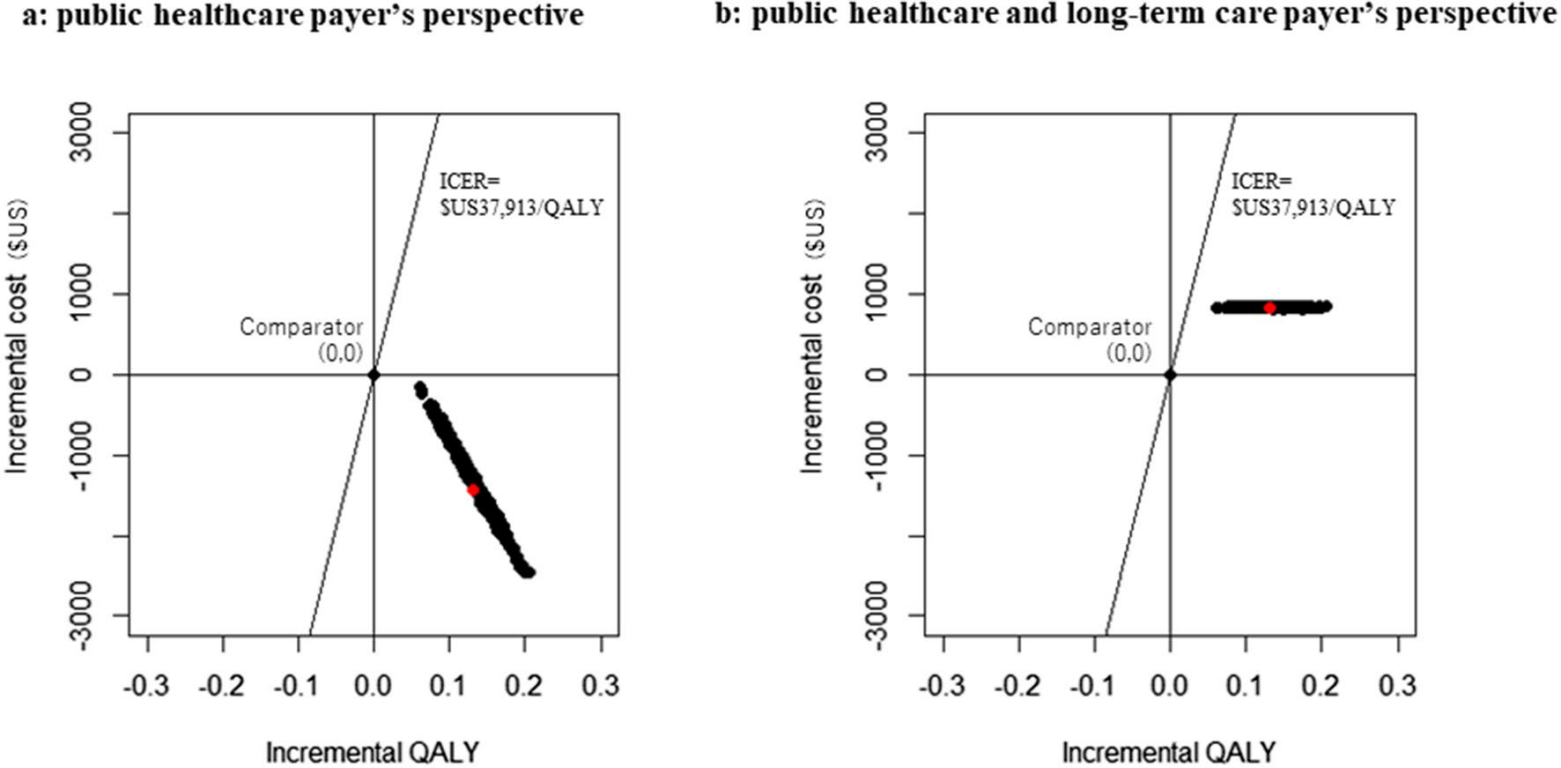
The results of probabilistic sensitivity analysis (**a**) from public healthcare payerʼs perspective and (**b**) from public healthcare and long-term care payerʼs perspective

The results of PSA were shown in Fig. 4. The ICERs were below the 5-million-yen willingness to pay with 100% probability both from public healthcare payerʼs perspective (Fig. 4a) and public healthcare and long-term care payerʼs perspective (Fig. 4b).

## Discussion

This study analyzed the cost-effectiveness of providing rehabilitation to acute stroke patients 7 days per week. From public healthcare payerʼs perspective The ICER at 5 years was $6339/QALY, lower than $37,913/QALY. Therefore, excellent cost-effectiveness of 7-days-per-week rehabilitation was expected. Kinoshita et al. analyzed the effectiveness of providing rehabilitation 7 days per week by comparing the 7-day and 5-/6-day groups. Their result was confirmed by including a large number of patients and by adjusting their results with various factors such as patient demographic information, time to admission after onset, and administration of recombinant tissue plasminogen activator [10]. This study clarified the economic aspects of a 7-days-per-week rehabilitation schedule for acute stroke patients.

The medical and long-term care system in Japan is guided by the national fee schedules [13]; thus, the cost analysis can be applied throughout the country. In this study, it was assumed that medical costs did not differ between the two groups, except for the frequency of rehabilitation. This result of excellent cost-effectiveness can be attributed to the fact that implementing a 7-days-per-week rehabilitation schedule does not cost much incrementally.

Long-term-care costs were lower for the 7-day group. The 7-days-per-week rehabilitation schedule was dominant, having better cost-effectiveness from public healthcare and long-term care payerʼs perspective. The decrease in the long-term care costs was attributed to the improved patient severity. Our result indicated that stroke patients can benefit from being assigned to lower care-needs levels, and subsequently, lower long-term care costs, by utilizing 7-days-per-week rehabilitation in the acute stage. The effect on patients suffering from post-stroke disability usually lasts for a lifetime. In Japan, approximately 26.9% of all long-term-care expenditure is attributed to stroke [29]. The Japanese guideline states that long-term care costs can be included when the effect of public long-term care costs is important. It is important to conduct cost-effectiveness analysis including long-term care costs of stroke treatments.

This research covered a sensitivity analysis considering uncertainty in the related parameters. The results showed that its cost-effectiveness remained at the high level, with the value of ICER was below the willingness to pay. The results indicate that 7-days-per-week rehabilitation for acute stroke patients was cost-effective even when considering the uncertainty of the results.

Murayama et al. reported that only 4 out of 25 subject hospitals were found to provide acute stroke patients with rehabilitation 7 days a week [9]. Initiating the 7-days-per-week rehabilitation schedule incurs costs for personnel (i.e., physicians, providers, and so on) who work on weekends or holidays, and administration costs, which can be a restraint for hospitals. The results of this study will be of importance in considering the optimal national fee in terms of cost-effectiveness and promoting the 7-days-per-week rehabilitation schedule. However, despite that costs for providing rehabilitation 7 days a week could be an obstacle from its implementation for hospitals, its cost-effectiveness from hospitals’ perspective is still unclear. Future research should focus on analysis from the perspective of hospitals.

This study analyzed the cost-effectiveness of the 7-days-per-week rehabilitation schedule for acute stroke patients to encourage the consideration of providing more cost-effective stroke treatments. Although the results here showed that 7-days-per-week rehabilitation was likely to be cost-effective, this study has several limitations. First, this analysis is mostly based on an observational database study by Kinoshita et al. [10], even though the study has large number of Japanese samples, and the efficacy of 7-days-per-week rehabilitation was evaluated after adjustment with related parameters. Therefore, there is still a possibility that the result can be biased by factors such as unobserved ones. Despite this limitation, the results of this study are of great importance when high-evidence study such as randomized controlled trials does not exist and is not likely to be conducted for this topic. Still, it is desirable that randomized controlled trails are conducted in the future to clarify more unbiased treatment effects of the intervention.

Second, since this study was from the healthcare payer’s perspective, this study did not consider opportunity costs; Yamaga et al. measured the cost of illness of stroke in Japan and included opportunity costs in their analysis [27]. Next, though there should be differences in the number of rehabilitation units, the relationship between rehabilitation time and patient severity has not been clear. Although a positive relationship between rehabilitation time and patient outcomes has been reported in the post-acute stage [31, 32], there is no consensus about the acute stage [33]. Some studies report that, by increasing intensity of rehabilitation in the acute stage, post-stroke severity in patients would be improved [3, 4]; however, Lauro et al. reported no significant effect of the same [34]. If the relationship is clarified, more detailed analysis on cost-effectiveness can be made available. Lastly, cost structures are different globally. Therefore, even though our methods are applicable to other countries, analyses should be conducted according to each country's system.

## Conclusions

This study examined the provision of cost-effective treatments for acute stroke patients, focusing on the cost-effectiveness of 7-days-per-week rehabilitation schedule. The estimated ICER was $6339/QALY, which was lower compared with the willingness-to-pay of 5,000,000 yen/QALY (approximately $37,913) from public healthcare payerʼs perspective, and 7-days-per-week rehabilitation schedule was dominant from public healthcare and long-term care payerʼs perspective. The results indicated that 7-days-per-week rehabilitation for acute stroke patients is likely to be cost-effective.

## Supplementary Information

**Additional file 1:** Calibration methods. Methods for the calculation to estimate the transition probabilities used in the Markov model were shown in the Additional file.

## Data Availability

The dataset(s) supporting the conclusions of this article is(are) included within the article.

## Abbreviations

ICER: Incremental cost-effectiveness ratio
QALY: Quality-adjusted life years
mRS: Modified Rankin Scale
QOL: Quality of life
PSA: Probabilistic sensitivity analysis
