# Supplementary material for: Cost-effectiveness of seven-days-per-week rehabilitation schedule for acute stroke patients
Source: Cost Eff Resour Alloc. 2023 Feb 1;21:12. doi: 10.1186/s12962-023-00421-3 (PMC9893661; doi:10.1186/s12962-023-00421-3)
Supplement: Supplementary file 1 — Additional file 1: Calibration methods. Methods for the calculation to estimate the transition probabilities used in the Markov model were shown in the Additional file. [file 12962_2023_421_MOESM1_ESM.docx]

Additional file: Methods for calibrating the Markov model

In this study, calibration was conducted to estimate the transition probability used in the Markov model. The Markov model consisted of three health states: independent (mRS0-2), severe (mRS3-5) and death (mRS6) (Figure 2 in the main text). Calibration was conducted using the Life Table of Japan [1], while the methods were according to those by Xie et al. [2]. It was based on the seven-step approach by Vanni et al. [3], described as follows.

Step1: Parameters included

The parameters targeted for calibration were determined as described by Xie et al. [2] (Table A1). The analysis period was divided into three phases (4-6, 7-12, 13-60 months).

Table A1: Targeted parameters

| Parameter | Period | Definition |
| --- | --- | --- |
| Rate_mRS02to35_ | 4-6 month, 7-12 month, 13-60 month | Annual disability rate of patients moving from mRS0-2 to mRS3-5 |
| Rate_mRS35to02_ | 4-6 month, 7-12 month | Annual disability rate of patients moving from mRS3-5 to mRS0-2 |
| Relative_Risk_mRS02to6_ | 4-6 month, 7-12 month, 13-60 month | Relative risk of mortality versus general population |
| Relative_Risk_mRS35to6_ | 4-6 month, 7-12 month, 13-60 month | Relative risk of mortality versus general population |

Rate_mRS35to02_ for 4–6 months and 7–12 was 0.455 and 0.188, respectively, and the monthly transition probabilities from mRS3–5 to mRS0–2 were 0.037 and 0.0156, respectively [2]. The rates were converted to monthly transition probabilities using Equation 1 [2]. The transition from mRS3–5 to mRS0–2 was assumed not to occur after 12 months.

Mortality (i.e., transition probability from mRS0–2 or mRS3–5 to mRS6) was estimated by multiplying the mortality rate of the general population by Relative_Risk_mRS02to6_ or Relative_Risk_mRS35to6_. The Life Table of Japan was used as the source of mortality for the general population [1].

$Transition probability=1-exp(-rate*t)$ Equation 1

t: time interval (in this study, 1month = 1/12)

Step 2: Calibration target

The results of the Oxford Vascular Study, used in the study of Xie et al. [2], were used as the calibration target because there was little data on the long-term outcomes of Japanese patients with acute stroke. This study used data relevant to moderate stroke patients (National Institutes of Health Stroke Scale (NIHSS) 4-10) because Kinoshita et al. [4], on which this study was based, focused on patients with similar stroke severity (NIHSS 5-6) (Table A2).

Table A2: Calibration target data [2]

| Period | mRS0-2 (%) | mRS3-5 (%) | mRS6 |
| --- | --- | --- | --- |
| 3 months | 30.3 | 47.5 | 22.2 |
| 6 months | 31.7 | 42.0 | 26.3 |
| 12 months | 29.9 | 36.5 | 33.6 |
| 24 months | 23.6 | 38.4 | 38.0 |
| 60 months | 15.4 | 28.6 | 56.0 |

Step 3: Measure of goodness-of-fit

For mortality, the absolute deviations between the observed mortality at a given time point (Table A2) and the calibration outputs of mortality were used to assess the goodness-of-fit. When the outputs were within acceptable ranges (i.e., ±1% from the observed mortality), the sum of squared errors (SSE) was calculated for the errors of the proportion of patients in the three states at all observation times in Table A1 [2]. The definition of SSE is described in Equation 2.

$SSE= \sum_{n=1} {(y_{i}-f\left( x_{i} \right))}^{2}$ (Equation 2)

n: the number of calibration targets (11, shown in Table A1)

yi: observed data at an observation time i
f(xi): calibrated output at an observation time i

Step 4-7: Parameter search strategy, acceptance criteria, stopping rule, and integrating calibration results into the economic model

These steps were also conducted as described by Xie et al [2]. We calibrated the parameters by grid search chronologically (i.e., we started with 4-6 months, and moved to 7-12 months after the ranges of parameters for 4-6 months were stable). After the ranges of the parameters were available, sets of parameters were generated using random search.
The acceptable ranges for mortality were < 1% at each time point, and those for the proportion of mRS0-2 patients were 95% confidence intervals of the observed data [2]. The process of random search process was repeated 100,000 times, and a set of parameters with the best goodness-of-fit (i.e. lowest SSE) was used to estimate the transition probabilities.

Calibration results:

The values of the set parameters with the best goodness-of-fit are shown in Table A3, and the estimated transition probabilities at each time point are shown in Table A4. The transition probabilities after 5 years were calculated based on the parameters for the 5th year while mortality was adjusted with the age-specific mortality rate obtained from the Life Table [1].

The proportion of patients in each health state and time cycle for the intervention and control groups is shown in Tables A5 and A6, respectively.

Table A3: The values of set parameters with best goodness-of-fit

| Parameter | Period | Definition |
| --- | --- | --- |
| Rate_mRS02to35_ | 4-6 months, 7-12 months, 13-60 months | 0.389, 0.285, 0.127 |
| Rate_mRS35to02_ | 4-6 months, 7-12 months, | 0.455, 0.188 |
| Relative_Risk_mRS02to6_ | 4-12 months, 13-60 months | 4.58, 2.12 |
| Relative_Risk_mRS35to6_ | 4-6 months, 7-12 months, 13-60 months | 14.83, 4.96 |

Table A4: Transition probabilities at each time period

| Period | mRS0-2 to mRS3-5 | mRS0-2 to mRS6 | mRS3-5 to mRS0-2 | mRS3-5 to mRS6 |
| --- | --- | --- | --- | --- |
| 4-6 months | 0.0319 | 0.00701 | 0.0372 | 0.02273 |
| 7-12 months | 0.0235 | 0.00701 | 0.0156 | 0.02273 |
| 13-24 months | 0.0105 | 0.00373 | 0 | 0.00872 |
| 25-36 months | 0.0105 | 0.00420 | 0 | 0.00982 |
| 37-48 months | 0.0105 | 0.00475 | 0 | 0.01111 |
| 49-60 months | 0.0105 | 0.00538 | 0 | 0.01259 |
| 61-72 months | 0.0105 | 0.00609 | 0 | 0.01424 |
| 73-84 months | 0.0105 | 0.00690 | 0 | 0.01613 |
| 85-96 months | 0.0105 | 0.00783 | 0 | 0.01831 |
| 97-108 months | 0.0105 | 0.00889 | 0 | 0.02078 |
| 109-120 months | 0.0105 | 0.01007 | 0 | 0.02354 |

Table A5: Proportion of patients in each health state and time point for the intervention group

| month | mRS0-2 | mRS3-5 | mRS6 |
| --- | --- | --- | --- |
| 6 | 48.7% | 43.3% | 8.0% |
| 12 | 44.1% | 40.2% | 15.7% |
| 24 | 38.4% | 37.0% | 24.6% |
| 60 | 22.1% | 33.7% | 44.2% |
| 120 | 7.2% | 15.4% | 77.4% |

Table A6: Proportion of patients in each health state and time points for the control group

| month | mRS0-2 | mRS3-5 | mRS6 |
| --- | --- | --- | --- |
| 6 | 39.4% | 52.1% | 8.5% |
| 12 | 37.0% | 46.1% | 16.9% |
| 24 | 32.2% | 41.3% | 26.5% |
| 60 | 18.5% | 35.1% | 46.3% |
| 120 | 6.1% | 15.1% | 78.8% |

References:

1. The Ministry of Labour, Health and Welfare. The 22th Life Table of Japan (FY2020). Available at <https://www.mhlw.go.jp/toukei/saikin/hw/life/22th/index.html> (Accessed on August 6, 2022)

2. Xie X, Lambrinos A, Chan B, Dhalla IA, Krings T, Casaubon LK et al. Mechanical thrombectomy in patients with acute ischemic stroke: a cost-utility analysis. CMAJ Open 2016;4(2):E316-25.

3. T Vanni, J Karnon, J Madan et al. Calibrating models in economic evaluation: a seven-step approach. Pharmacoeconomics. 2011 Jan;29(1):35-49.

4. Kinoshita S, Momosaki R, Kakuda W, Okamoto T, Abo M. Association Between 7 Days Per Week Rehabilitation and Functional Recovery of Patients With Acute Stroke: A Retrospective Cohort Study Based on the Japan Rehabilitation Database. Archives of Physical Medicine and Rehabilitation. 2017; 98: 701-06.
